# Tourism’s long- and short-term influence on global cities’ economic growth: The case of Hong Kong

**DOI:** 10.1371/journal.pone.0275152

**Published:** 2022-09-29

**Authors:** Andy C. L. Tai, David W. H. Wong, Harry F. Lee, W. Qiang

**Affiliations:** 1 Division of Business and Hospitality Management, College of Professional and Continuing Education, The Hong Kong Polytechnic University, Hong Kong, Hong Kong; 2 Department of Management, The Hang Seng University of Hong Kong, Hong Kong, Hong Kong; 3 Department of Geography and Resource Management, The Chinese University of Hong Kong, Hong Kong, Hong Kong; Universiti Malaysia Sabah, MALAYSIA

## Abstract

This research examines how tourism development has impacted economic growth in a global city–Hong Kong. A large body of research has investigated national tourism-led growth in developed and developing countries. However, many such studies have overlooked how policies aimed at fostering the development of tourism affect the local economic development of global cities. The Chinese and Hong Kong governments liberalized their visa policies with the launch of the Individual Visit Scheme in 2003. Such liberalization has led to significantly more tourist arrival from China. Our autoregressive distributed lag model of tourism-related data from 2003 to 2019 provides strong evidence that more tourism can spur short-run economic growth. Yet, such tourism can lead to uncertain effects on local economic development in the longer run. Hong Kong’s transient tourism-led growth has almost entered the stagnation stage of the Tourism Area Life Cycle model. During such stagnation, jurisdictions like Hong Kong can expect limited long-term economic growth from their tourist sector. Our findings thus sound a warning for global cities looking to tourism to sustain longer-term economic growth.

## 1. Introduction

The travel and tourism industry represents one of the most extensive and fastest-growing sectors worldwide. Such tourism thus contributes significantly to national and regional economic development in many developed and developing economies. The World Travel and Tourism Council estimates tourism accounted for about 10% of global gross domestic product (GDP) and global employment in 2019. Yet, the sector lost a bit more than US$9 trillion in 2019 and less than US$5 trillion in 2020 as a result of the COVID-19 pandemic. Such losses trimmed almost 4% of GDP off the global economy. Global employment in the sector fell about 19% from 334 million in 2019 to 2020 [1]. The COVID-19 crisis devastated the travel and tourism industry, posing severe challenges for the recovery of the global economy.

Hong Kong’s economy has been particularly prone to the COVID-19 pandemic’s economic effects. According to the Hong Kong Tourism Board, international tourist arrivals in Hong Kong grew from 13.6 million in 2000 to 55.9 million in 2019 [2]. Revenue from such tourism receipts rose from US$7.9 billion in 2000 to US$32.8 billion in 2019 [3]. The signing of the Individual Visitor Scheme between the Mainland and Hong Kong in 2003 exacerbated these trends. Mainland tourist arrivals multiplied 5 times from 8.4 million in 2003 to 2019. Mainland tourists accounted for about 78% of Hong Kong’s tourism market share. Tourism—especially from mainland China—became to form one of Hong Kong’s central economic pillars.

For decades, policymakers in places like Hong Kong have considered tourism an engine of economic growth. However, the chicken-and-egg problem affects tourism’s effect on economic growth or *visa-versa*. Fast-growing metropolises attract far more tourists than stagnant ones. According to Solow’s neoclassical growth model, an aggregated production function approach has been adopted and postulates technological change as an exogenous variable [4]. Yet, a city’s touristic luster develops independently and exogenously from decisions about deploying capital in the most productive way. Thirty years later, economists like Mankiw, Romer, and Weil, and Romer saw the potential such tourism could bring [5, 6]. Tourism brings talented innovators, business people, and touristic places attract research interest. Such ‘human capital’ forms part-and-parcel with the endogenous technological innovation that drives Solow’s capital into scenic world cities. In recent years, theoreticians and econometric modelers have directly incorporated tourism into their models of economic growth. Tourism brings employment opportunities, foreign exchange earnings, and infrastructure improvements (among other things); positively contributing to a country’s/region’s economic growth and development [7]. Tourism also promotes connections between sectors—adding economies of scope and helping redistribute growth to lagging regions [8]. Asset bubbles, the increased cost of living, environmental degradation, and the over-exploitation of natural resources represent clouds to these silver linings in Hong Kong and other global cities [9].

Four major hypotheses have dominated the literature’s discussion about the association between tourism and economic growth [10]. First, the *tourism-led growth hypothesis* has recognized the catalytic effect of tourism on the economic growth process in many countries/ regions. It has argued for tourism’s direct contribution to components of GDP like the hotels/ hospitality sector, travel agents, passenger transport, and other leisure/ recreational services [11]. Unlike other sectors like manufacturing or finance, governments and/or public-private consortia must work together to increase investment in infrastructural development [12]. Second, the *economy-driven tourism growth hypothesis* posits a country/ region’s economic growth can strengthen the tourism sector. It indicates unidirectional causality from economic development to tourism, not *vice-versa*. More business travelers and upgrades to electric/ water mains and roads occur only when the local business expands enough to demand and pay for these services [13]. Third, the *feedback hypothesis* postulates bi-directional causality between tourism and economic development. Developing tourism and economic growth jointly determine each other [14, 15]. Finally, the *neutrality hypothesis* argues that tourism has no significant effect on economic growth [16].

Numerous dynamic models have sought to move past these simplistic views of tourism-led growth. Butler’s Tourism Area Life Cycle (TALC) model, for example, depicts the evolution of a tourist area from its discovery to its final stage [17]. A tourism area’s S-shaped growth on a graph of time versus growth passes through the six stages of exploration, involvement, development, consolidation, stagnation, and decline (or, in some cases, rejuvenation). Some researchers point to the progressively increasing growth rates of tourist arrivals in a specific tourist area during the exploration, involvement, and development stages of a tourist area [18, 19]. Decreasing tourist visits characterize the consolidation and stagnation stages of the area’s life cycle. In all stages, a tourist area’s economic growth goes hand-in-hand with its tourism development.

The literature, though, still leaves two questions unanswered. First, how does the tourism-economic growth relationship work for global cities? According to Globalization and World Cities Research Networks, global cities are important sites for foreign direct investment and take a pivotal role in production, finance, and advanced producer service to facilitate the operation of multinational corporations. New York, London, Singapore, Hong Kong, Paris, Beijing, Shanghai, Tokyo, and Madrid are typical global cities. These areas have large, urbanized populations working and living in a diversified economy [20]. If many researchers examine the tourism-growth nexus at the international, regional, and country levels, they leave the push and pull of global city factors completely unexplored [15, 21]. Second, how has empirical or econometric methodology sorted out whether global cities encourage tourism? Time series arguments like Granger causality cannot identify the source of this growth. These methods also over-simplify the interaction between these factors [22]. Even if they could perform these feats, existing models cannot identify the short-term versus long-term effects of tourism on economic growth and *visa-versa* [23]. Authors like Song and Wu have called for their peers to comprehensively review the still nebulous association between tourism development and economic growth [24]. The results would allow policymakers to promote regional economic development with more effective tourism marketing and policy decisions.

Looking specifically at Hong Kong, we systematically examine how its economic growth responded to tourism after the 1997 Handover. Hong Kong represents a prime example of a renowned global city embedded with other prominent, factor-intensive, and trade-heavy metropolitan areas [25]. Understanding the growth-tourism nexus in Hong Kong thus teaches us something about this nexus in other global cities. The 2003 Individual Visit Scheme (IVS) also offers a large-scale natural experiment—or event where tourism changed independently of economic factors in any particular region. We use an autoregressive distributed lag (ARDL) model on several variables ranging from 2003 to 2019. The ARDL model will thus help us assign short-run and long-run effects on the role of tourism (and other factors) in Hong Kong’s economic development over the roughly 17-year period. The analysis will allow us to answer three questions left by gaps in the literature we cite above. First, does the TALC model apply to Hong Kong in the early part of this century? Second, what influence has tourism had on Hong Kong’s economic growth? Third, do short- and long-run factors influence how tourism development and economic growth evolve over time?

Our study contributes to the tourism-growth literature in three ways. First, our research provides new insights into how existing tourism and economic growth evolve in a global city. Many researchers—focused on countries or popular regions—have overlooked the large metropolitan regions that drive much global growth. Second, our study allocates the effects of tourism and growth in short and long-run terms. We find that tourism does not affect contemporaneous economic growth in the short run. The data also show a statistically significant one-quarter lagged effect on tourism development on economic growth. These results suggest that policies aimed at promoting tourism in places like Hong Kong have only transient effects. Third, and finally, recalling the TALC model, Hong Kong’s stagnation period in its tourism has resulted in short-term positive economic growth, while long-term growth spurts have not made up for. These findings argue that policymakers like Hong Kong can lean on tourism policies to juice growth—particularly during down-turns like those caused by the recent COVID-19 pandemic.

Our article has six sections. Section 2 analyzes Hong Kong’s current tourism sector. Section 3 outlines our methodology and our model specifications. Section 4 describes the data we used and our variable selection. Section 5 presents and discusses our empirical results. The concluding section draws together conclusions and presents implications from our study.

## 2. Hong Kong’s tourism sector

**Fig 1:**
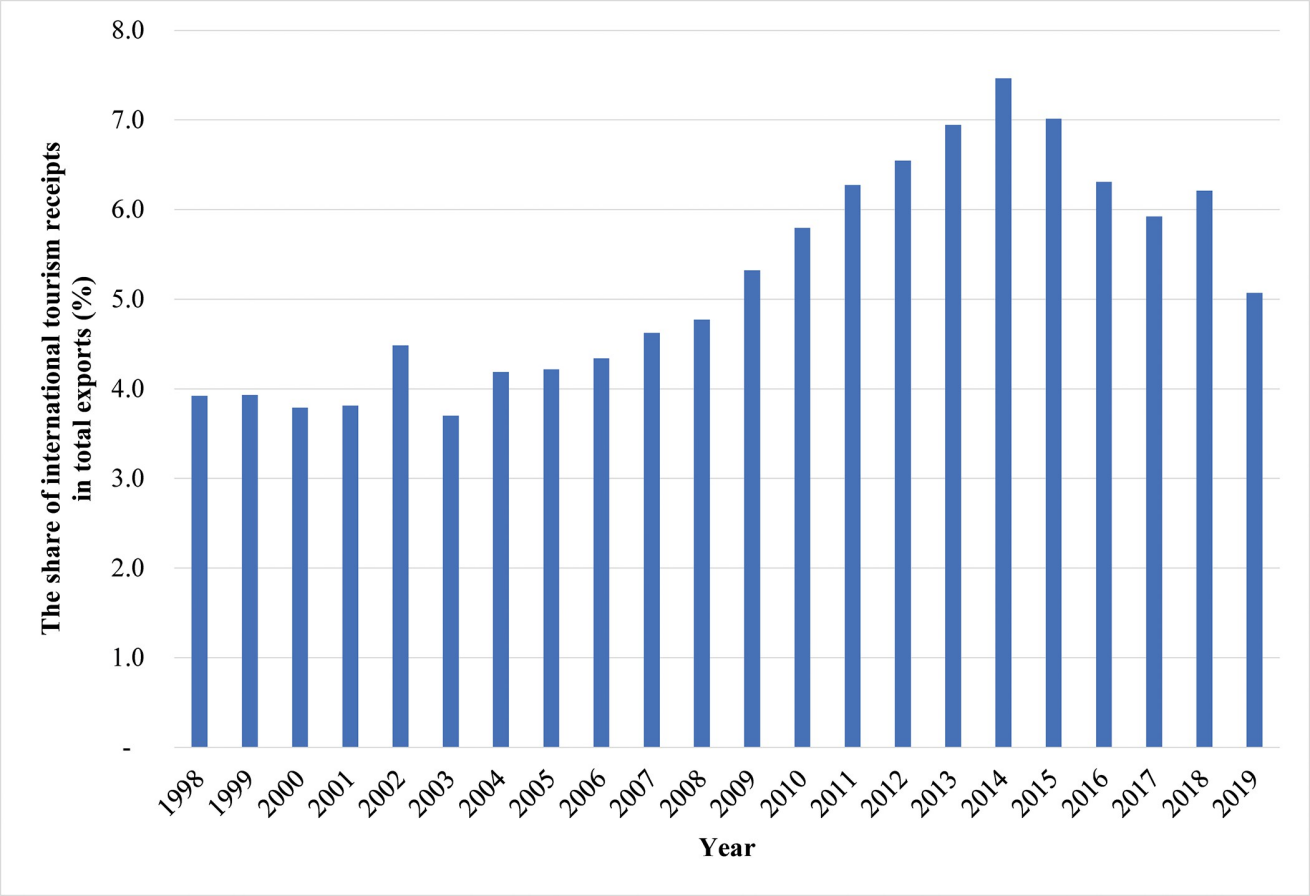
The share of international tourism receipts in Hong Kong’s exports, 1998–2019. Data source: The World Bank. https://data.worldbank.org/indicator/ST.INT.RCPT.XP.ZS?locations=HK.

**Fig 2:**
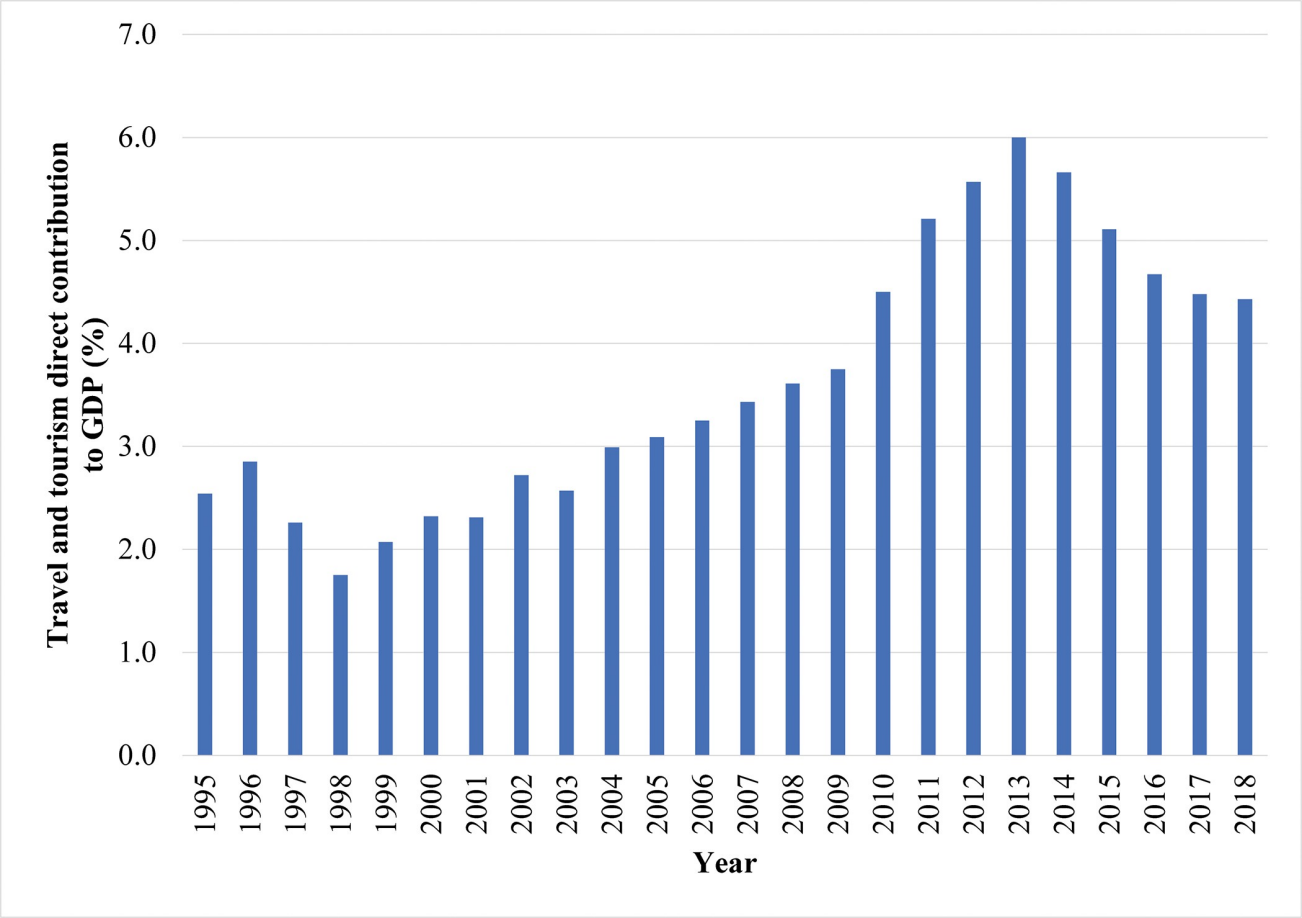
The contribution of the travel and tourism sector to Hong Kong’s gross domestic product, 1995–2018. Data source: World Travel & Tourism Council. https://tcdata360.worldbank.org/indicators/tot.direct.gdp?country=HKG&indicator=24648&countries=BRA&viz=line_chart&years=1995,2028.

Over the past two decades, the tourism industry gradually became one of the strategic sectors in Hong Kong’s economy. According to Fig 1, the share of international tourism receipts in total exports increased from 3.9% in 1998 to 7.5% in 2014 and then declined to 5.1% in 2019. Moreover, based on Fig 2, the contribution of travel and tourism to Hong Kong’s GDP grew from 2.5% in 1995 to about 6.0% in 2013 and then dropped to 4.4% in 2018. The data trends in the two figures match closely.

**Fig 3:**
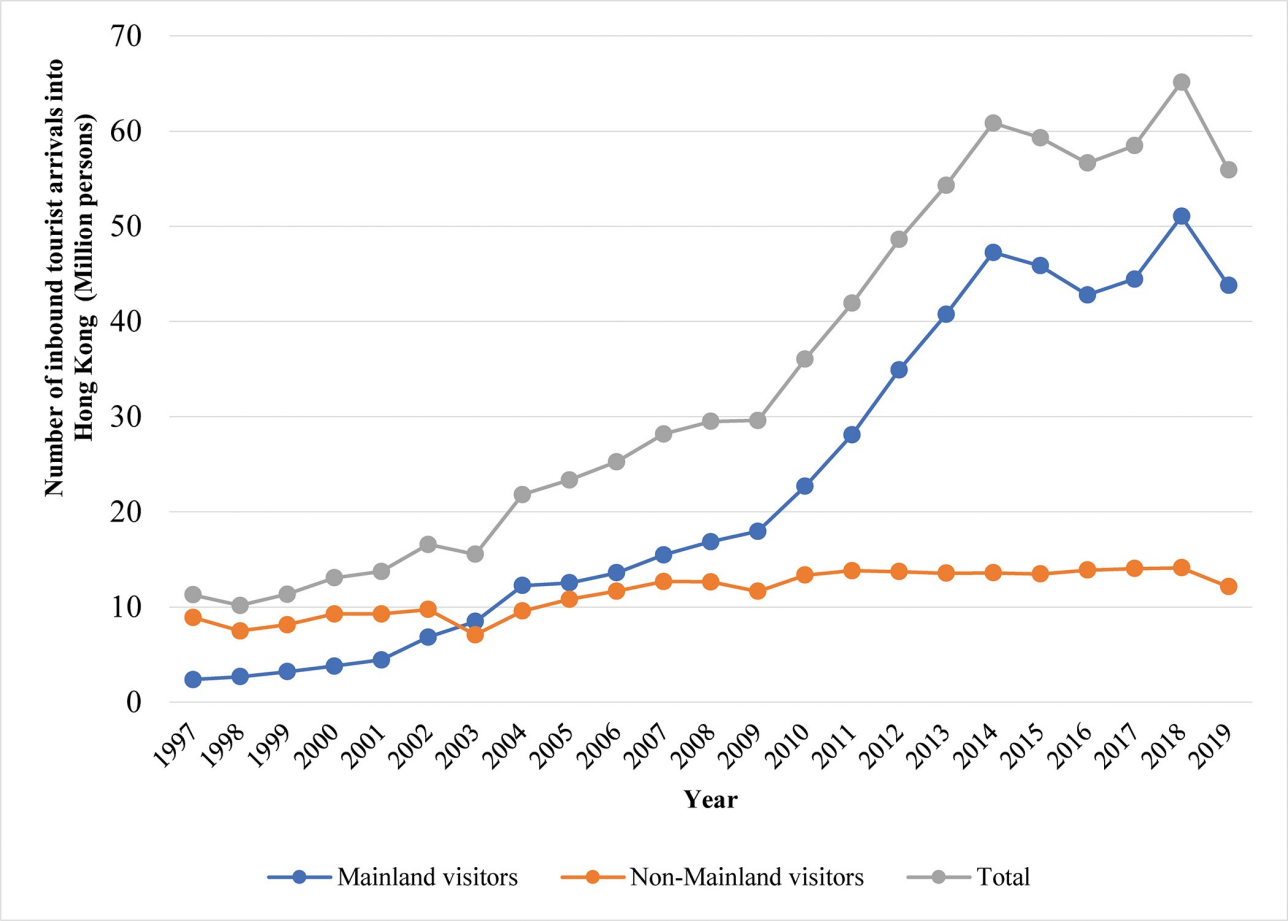
The number of inbound tourism arrivals into Hong Kong, 1997–2019. Data source: Hong Kong Annual Digest of Statistics. https://www.censtatd.gov.hk/en/EIndexbySubject.html?scode=100&pcode=B1010083.

Mainland visitors to Hong Kong have exploded since the Handover in 1997. Fig 3 shows the number of inbound tourist arrivals into Hong Kong from 1997 to 2019. Before 1997, Mainlanders could only visit Hong Kong by applying for business visas or joining organized group tours. From 1997 to 2002, Mainland visitor numbers steadily increased from 2.4 million to 6.8 million. The Severe Acute Respiratory Syndrome (SARS) epidemic in 2003 disrupted this growth. In response, government authorities on both sides of the border set up the IVS in July. The Scheme allowed Mainland visitors to travel to Hong Kong for up to seven days at a time. They could make only one or two trips per year under the Scheme and originate from only four cities in the Guangdong province (the province abutting Hong Kong). Mainland visitors to Hong Kong under the IVS scheme rose gradually from 8.5 million in 2003 to 18.0 million in 2009 and 22.7 million in 2010.

Following the global financial crisis in 2009, the Shenzhen government allowed its permanent residents to visit Hong Kong multiple times under a modification to the IVS scheme. Such a visitor received an M-permit under the IVS scheme, with the M-permit endorsement granting the bearer the right to enter Hong Kong multiple times during one year. By 2015, Mainland tourist arrivals hovered at around 45.8 million people.

The growth of illegal black-market trade across the Shenzhen-Hong Kong border encouraged the authorities to modify the M-permit regime in 2015. In April 2015, government leaders announced the M-permit’s replacement with an Individual Visit Endorsement, allowing one weekly trip. By 2020, the mainland government extended the IVS scheme to 49 cities in 18 provinces [26]. Yet, the number of Mainland visitors remained relatively stable during this period. Mainland tourist arrivals from 2014 to 2018 only increased by about 3.8 million people. Social unrest in Hong Kong in 2019 led to a drop in Mainland visitors to about 44 million.

The introduction of the IVS scheme had a noticeable effect on the type and number of tourists/ visitors to Hong Kong. Non-Mainland visitors to Hong Kong rose from 8.9 million in 1997 (the year of Hong Kong’s Handover) to 12.6 million in 2008 (the start of the financial crisis). Such a rise vastly exceeded the increase in Mainland visitors until the introduction of the IVS scheme in 2003. The number of non-Mainland visitors oscillated between 13.3 million and 14.1 million from 2010 to 2018. By the end of 2019, non-Mainland visitors declined to 12.1 million. Such numbers make up only about one-quarter of the number of Mainland tourists. Since the COVID-19 pandemic, inbound tourism has almost halted from January 2021 [27].

In Butler’s TALC model, Hong Kong’s current tourist figures lie at the stagnation stage of the city’s tourism life cycle curve. The general reduction in tourist arrivals and the decline of the travel and tourism sector’s contribution to Hong Kong’s GDP after 2018 implies Hong Kong’s tourism infrastructure has reached its carrying capacity.

**Fig 4:**
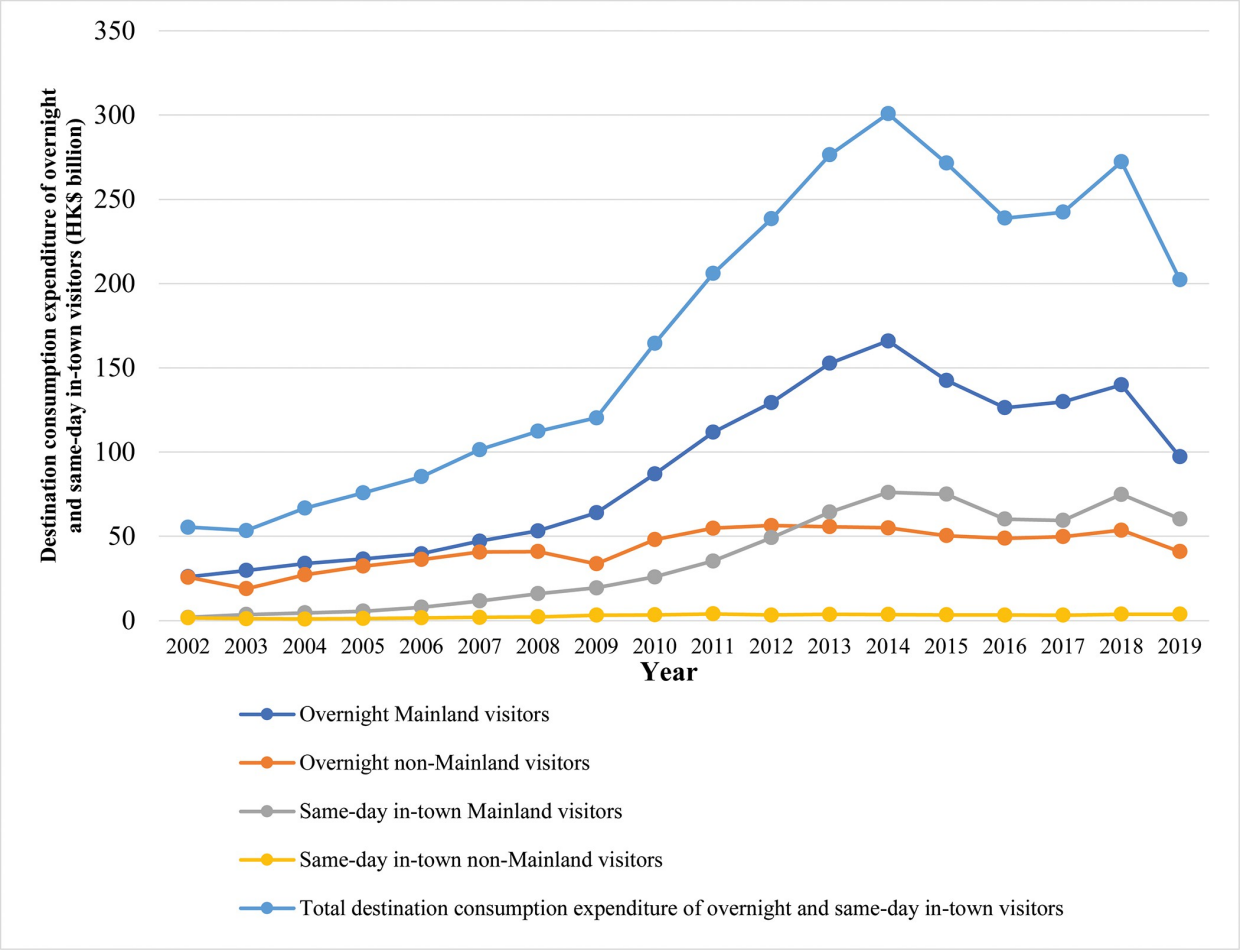
Tourist spending by same-day and overnight visitors to Hong Kong, 2002–2019. Data source: Hong Kong Annual Digest of Statistics. https://www.censtatd.gov.hk/en/EIndexbySubject.html?scode=100&pcode=B1010083.

Spending in Hong Kong by Mainland and non-Mainland tourists varies, depending on whether they return to the Mainland on the same day or spend at least one night in Hong Kong. As shown in Fig 4, spending by Mainland visitors to Hong Kong grew sharply from HK$26.1 billion in 2002 to HK$166.0 billion in 2014. Such growth represents a 41% compounded annualized growth rate in such spending until the end of 2014. In 2015, such spending gradually declined from HK$142.6 billion to HK$126.3 billion in 2016, bouncing back to HK$139.9 billion in 2018. By 2019, such spending plunged again to HK$97.2 billion.

Spending by non-Mainland visitors at least one night in Hong Kong differed significantly from the trends described previously. Such spending increased from HK$25.7 billion in 2002 to HK$41.0 billion in 2008. Such spending slightly dropped to HK$33.7 billion in 2009 and hovered between HK$48.1 billion in 2010 and HK$53.7 billion in 2018. Reflecting the first results of the pandemic, tourist spending in Hong Kong by this stratum of tourists plummeted to HK$41.0 billion in 2019.

Spending by Mainland day-trippers skyrocketed from HK$2.0 billion in 2002 to HK$76.1 billion in 2014. Such spending then fell from HK$75.1 billion in 2015 to HK$59.5 billion in 2017. Since then, their spending has chaotically bounced from HK$74.9 billion in 2018 to HK$60.4 billion in 2019. Day-trippers’ spending thus mirrors the trends in spending by Mainland tourists spending at least one night in the city. However, spending by non-Mainland visitors bucked these trends. Whether day-tripping or spending the night in Hong Kong, their spending remained remarkably stable from 2002 to 2019. Such spending stayed between HK$1.6 billion (in 2002) and HK$3.8 billion (in 2019).

## 3. Methodology and model specification

We use a Cobb-Douglas production function to examine whether international tourists’ arrivals would promote a city’s economic growth. In this function, two physical inputs—capital and labor—combine to create an economic output (as shown in Eq 1). Our data come from the Hong Kong Annual Digest of Statistics and Hong Kong Monthly Digest of Statistics and cover the period from the third quarter of 2003 to the third quarter of 2019.

$$Y={\mathrm{AK}}^{\alpha }L^{\beta }$$ (1)

Real GDP *Y* emerges from total factor productivity *A*, the use of physical capital *K*, and labor *L*. The parameters α and β determine the relative impact of physical capital and skill-augmented labor on such output.

Following authors like Algaeed, Durbarry, Jin, Mankiw, Romer, and Weil, we incorporated other variables into our equation to make our model more realistic [5, 28–30]. We specifically include human capital development *H*, the market capitalization of Hong Kong Growth Enterprise Market (GEM) *G* in a time period *t* (represented as a quarter between 2003 and 2019). GEM is a board of the Stock Exchange of Hong Kong serving the need to attract listings by small and mid-sized technology firms and start-ups. GEM’s market capitalization can reflect the levels of major business activities of those firms in Hong Kong. We also include the number of international tourist arrivals *TA*, allowing for errors exhibited in certain traits. Based on the above theoretical arguments, we specify an econometric equation as follows:

$$Y_{t}=a+\lambda_{1}K_{t}+\lambda_{2}L_{t}+\lambda_{3}H_{t}+\lambda_{4}G_{t}+\lambda_{5}{\mathrm{TA}}_{t}+\varepsilon_{t}$$ (2)

where t denotes year t from the third quarter of 2003 to the third quarter of 2019; Y_*t*_ represents the natural logarithm of real GDP; K_*t*_ indicates the natural logarithm of physical capital; L_*t*_ is the natural logarithm of labor input; H_*t*_ represents the natural logarithm of human capital development; G_*t*_ stands for the natural logarithm of gaming revenue; TA_*t*_ represents the natural logarithm of the number of international tourist arrivals; λ_1_, λ_2_, λ_3_, λ_4_, and λ_5_ are the parameters and ε_*t*_ is an error term.

The ARDL approach makes the relationship shown in Eq 2 more dynamic. Specifically, we perform the data transformation by log-differencing all variables for econometric reasons and make Eq 2 allow us to regress lagged values of the dependent and independent variables. Results related to short lags describe short-term effects, and a slight mathematical manipulation of the equation allows us to find any possible long-term equilibria. The Error Correction Model (ECM)–a simple recombination of the equation shown above—demonstrates how quickly the variables return to their long-term equilibria. Compared to conventional estimation techniques, such as Granger causality, the ARDL method can evaluate the short-run and long-run relationship between tourism development and economic growth. As such, the ARDL method is more relevant in our study [31–33]. Numerous studies have also used similar approaches in contexts like ours [23–34].

Which lags should we choose? And will the variables react to each other in the same time period? In other words, are our variables cointegrated (meaning we do not need to remove past information about our variables to do our analysis)? By applying the ARDL technique, we further develop our following model. We use Augmented Dickey-Fuller (ADF) and Philips-Perron (PP) unit root tests to estimate each variable’s level value, first-order difference, and second-order difference over time. The constant *a* does not change over time, the error term ε equals zero over a short span of time and *θ* represents the effect that past output has on current output. If Δ represents a change in a variable between two immediate periods; thus, Δy_*t*_ represents the difference between y in time *t* and time *t-1*. Eq 3 shows the simplest one-period difference, whereas Eq 4 shows the difference with *i* periods in the past. Variables with bars over them represent constants.


$$\Delta y_{t}=\bar{a}+\Delta y+\Delta k_{t}+\Delta l_{t}+\Delta h_{t}+\Delta g_{t}+\Delta {\mathrm{ta}}_{t}+\varepsilon$$ (3)

If we allow for Δ over many periods, we could have θ_*t-1*_y_*t-1*_ + θ_*t-2*_y_*t-2*_ and so forth on the current y_*t*_. Each theta represents a separate time effect. Variables with short lags represent short-term effects, and those with long-term lags represent longer-term effects. Eq 4 expresses this mathematically—with a sub-set of lag terms representing these short-term effects and a bar over the top to indicate a stable, equilibrium value.


$$y_{t}=\bar{a}+\bar{\theta }y+\bar{\alpha }k_{t}+\bar{\beta }l_{t}+\bar{\gamma }h_{t}+\bar{\eta }g_{t}+\bar{\phi }{\mathrm{ta}}_{t}+\sum_{t=1}^{n}\theta_{t-i}y_{t}+\sum_{t=1}^{n}\alpha_{t-i}k_{t}+\sum_{t=1}^{n}\beta_{t-i}l_{t}+\sum_{t=1}^{n}\gamma_{t-i}h_{t}+\sum_{t=1}^{n}\eta_{t-i}g_{t}+\sum_{t=1}^{n}\phi_{t-i}{\mathrm{ta}}_{t}+\mu$$ (4)

If our variables affect each other in the long run and thus exhibit co-integration, we will observe the same long-term and short-term parameters described in the equation above. The adjustment toward the long-run values represents the error-correction part of our model. We do not choose our period *i* arbitrarily. We look at the lags with the best Akaike Information Criteria (AIC). We checked the usual assumptions behind time series models, using the relevant diagnostic tests when we were unsure about the good behavior of some of our variables. We found no issues that jeopardized our procedure or required changes to the data.

## 4. Data and variable selection

We suppose real GDP develops according to the production function we have described. We use the number of international tourist arrivals as one of the explanatory variables to test the effect of tourism development on economic growth. As a city’s economy grows in line with the development of its tourism resources and visitors, we hypothesize that tourism positively affects economic growth.

To examine this tourism-growth relationship in Hong Kong, we control for a number of factors. First, we include the amount of physical capital used in production as gross fixed capital formation. Second, we include employment figures to control the labor used in production. Third, we include the number of secondary school graduates as our proxy for human capital development. Hong Kong is an international financial hub. We use the GEM’s market capitalization to control the business activities of small and mid-sized technology firms and start-ups listed in Hong Kong. The extent of market capitalization represents our final control variable. These control variables thus represent the variables we described in Eqs 3 and 4 above.

## 5. Results and discussion

Before analyzing our results, we need to look at the validity of our regression. Table 1 presents the descriptive statistics for our entire data set. These statistics indicate that our variables follow the Gaussian distributions required for linear regression. We specifically show the skewness and kurtosis test results for our variables. The ARDL bounds approach to cointegration deployed in this study will minimalize the effects of some outliners, making our measures and inferences more robust. Table 2 shows the correlation between our variables. The correlation matrix reveals that the number of international tourist arrivals (*ta*) and economic growth (*y*) are positively correlated at a 1% significant level, providing preliminary evidence to support the tourism-growth nexus. More importantly, no correlation coefficients appear high enough to warrant concerns about multicollinearity between our variables.

**Table 1:** Descriptive statistics for the whole data set.

	y	k	l	h	g	ta
**Mean**	0.014	0.011	0.002	-0.006	0.011	0.031
**Median**	0.033	0.016	0.002	0.000	0.023	0.007
**Maximum**	0.113	0.176	0.013	0.031	0.452	0.987
**Minimum**	-0.131	-0.188	-0.009	-0.113	-0.485	-0.336
**Standard deviation**	0.063	0.087	0.004	0.021	0.181	0.149
**Skewness**	-0.387	-0.541	0.183	-3.244	-0.352	4.005
**Kurtosis**	-1.081	0.083	0.347	12.837	1.051	26.845
**Observation**	65	65	65	65	65	65

**Table 2:** Correlation matrix of each variable.

Variables	y	k	l	h	g	ta
**y**	1					
**k**	0.489***	1				
	(0.056)					
**l**	0.109	0.101	1			
	(0.063)	(0.087)				
**h**	0.179	0.192	0.256**	1		
	(0.063)	(0.086)	(0.004)			
**g**	0.088	0.087	0.261	0.074	1	
	(0.063)	(0.087)	(0.004)	(0.021)		
**ta**	0.485***	0.178	0.146	0.034	0.014	1
	(0.056)	(0.086)	(0.004)	(0.021)	(0.181)	

Notes

* Indicates significance at 10% level.

** Indicates significance at 5% level.

*** Indicates significance at 1% level.

**Fig 5:**
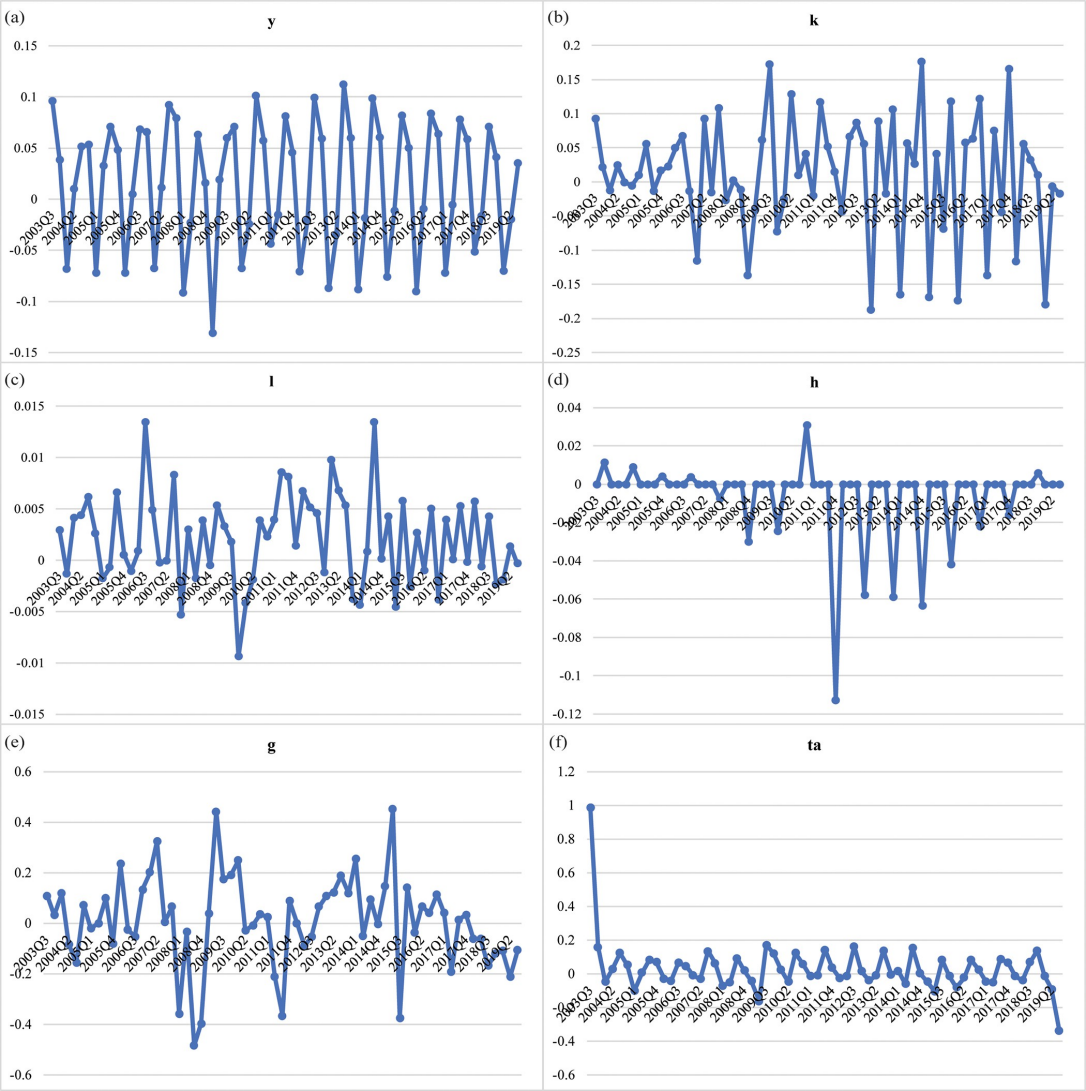
Trend charts of real GDP (*y*), physical capital (*k*), labor (*l*), human capital development (*h*), stock market capitalization of the Growth Enterprise Market (*g*), and the number of international tourism arrivals (*ta*).

Besides, non-stationarity (when variables are not cointegrated) represents a first problem that can invalidate our regression results. Fig 5A–5F show the difference in our variables’ natural logarithmic form over our period. As expected from these trends, our variables likely contain unit roots (and are thus integrated). Table 3 reports the results of the ADF and PP tests we described earlier–looking for unit roots. All our variables show statistically significant integration over one period.

**Table 3:** Unit root test results.

Variables	ADF test value	PP test value
At level		
y	-4.119***	-9.743***
k	-1.889	-4.396***
l	-2.709	-2.302
h	-2.212	-2.186
g	-2.444	-2.379
ta	-0.969	-8.133***
At first difference		
Δy	-4.341***	-13.305***
Δk	-3.752**	—19.637***
Δl	-3.912**	-8.044***
Δh	-8.653***	-8.716***
Δg	-6.245***	-6.229***
Δta	-7.656***	-23.227***

Notes: ADF denotes Augmented Dickey-Fuller test for unit root, and PP represents Phillips and Perron test for unit root.

* Indicates significance at 10% level.

** Indicates significance at 5% level.

*** Indicates significance at 1% level.

To examine the co-integrated relationships among all variables, we performed the bounds test developed by Pesaran, Shin, and Smith [33]. Table 4 presents the bounds test for co-integration. Since the calculated value of the F-statistic (51.481) is greater than the upper bounds critical value (4.764) at a 1% significant level, all variables are co-integrated. Concurrently, our result of co-integration confirms the existence of the short- and long-run associations among the variables in the ARDL model specification.

**Table 4:** Bounds test for co-integration.

F-Bounds Test		Null hypothesis: No levels relationship		
Test Statistic	Value	Significant	I(0)	I(1)
			Asymptotic: n = 1,000	
**F-statistic**	**51.481**	10%	2.26	3.35
K	5	5%	2.62	3.79
		2.5%	2.96	4.18
		1%	3.41	4.68
Actual Sample Size	63		Finite Sample: n = 65	
		10%	2.39	3.54
		5%	2.84	4.09
		1%	3.78	5.31
			Finite Sample: n = 60	
		10%	2.39	3.57
		5%	2.82	4.09
		1%	3.78	5.34

As previously mentioned, we used the AIC to determine the optimal lag length for each variable. The best information criteria of tourism development for lag times are two periods. After applying the appropriate lags and rearranging our equation to fit the ECM format, we estimated the short and long-term effects between tourism development and economic growth. Table 5 reports whether the data exhibited a long-run estimate. According to our ARDL model, the first variable shows one period lag while the remaining variables demonstrate two periods lag (or ARDL (2,1,2,2,2,2)). Our results show tourism development has no causal link with economic growth in the long run (i.e., in the same period), which has the same result reported by Tang [16]. Still, tourism development has a positive and statistically significant influence on economic growth after a one-period lag. For example, a 1% increase in tourism development today leads to a 0.205% rise in economic growth tomorrow. Corroborating authors like Oh and Tugca [7, 8], our results show that the development of tourism in Hong Kong likely contributed to transitory economic growth in the area. However, such effects do not persist.

**Table 5:** Long run ARDL estimate.

Variables	Coefficient	Standard error	p-values
**y(-1)**	**-0.354** ***	**0.089**	**0.000**
**y(-2)**	**-0.689** ***	**0.075**	**0.000**
k	0.036	0.049	0.465
k(-1)	0.126	0.048	0.012
l	-0.946	0.831	0.261
**l(-1)**	**3.124** ***	**0.852**	**0.001**
**l(-2)**	**-1.772** **	**0.856**	**0.044**
h	-0.016	0.173	0.929
h(-1)	-0.009	0.176	0.958
**h(-2)**	**0.372** **	**0.167**	**0.031**
G	0.002	0.017	0.899
g(-1)	0.027	0.019	0.154
**g(-2)**	**0.042** **	**0.019**	**0.033**
ta	0.064	0.047	0.175
**ta(-1)**	**0.205** ***	**0.072**	**0.007**
ta(-2)	-0.042	0.026	0.108
**Constant**	**0.021** ***	**0.004**	**0.000**

*Indicates significance at 10% level.

**Indicates significance at 5% level.

***Indicates significance at 1% level.

According to Table 6, the ECM produced an error correction term, which was -0.902 and significant at the 1 percent level, implying a short-run relationship prevails between the dependent variable and the regressors. Looking at the error correction side of our model, roughly 90% of a shock to tourism persisted beyond a single quarter. Changes in tourism definitely affect GDP growth—just as such, growth affects how tourism changes in Hong Kong from quarter to quarter. Nevertheless, even if they persist in small amounts, the relationships between the growth in tourism and GDP continue in the longer run.

**Table 6:** Short run ARDL estimate.

**Variables**	**Coefficient**	**Standard error**	**p-values**
Δy(-1)	0.008	0.179	0.734
Δy(-2)	-0.412	0.127	0.966
**Δk**	**0.092** ***	**0.054**	**0.002**
**Δk(-1)**	0.039*	**0.058**	**0.097**
Δl	-1.137	1.007	0.265
Δl(-1)	1.129	1.084	0.303
**Δl(-2)**	**-2.595** ***	**0.912**	**0.007**
Δh	-0.013	0.187	0.945
Δh(-1)	0.279	0.254	0.278
**Δh(-2)**	**0.541** ***	**0.191**	**0.007**
Δg	-0.014	0.021	0.478
Δg(-1)	0.021	0.023	0.361
Δg(-2)	0.018	0.021	0.399
**Δta**	**0.093** *	**0.054**	**0.096**
**Δta(-1)**	**0.168** **	**0.074**	**0.027**
Δta(-2)	-0.009	0.033	0.787
**ECT**	**-0.902** ***	**0.271**	**0.002**

*Indicates significance at 10% level.

**Indicates significance at 5% level.

***Indicates significance at 1% level.

We checked the robustness of our model by looking at testing for all the usual problems. The Breusch-Godfrey serial correlation Lagrange Multiplier test found no autocorrelation in the residual terms. A Breusch-Pagan-Godfrey test found no heteroskedasticity in our model’s residual terms. A Ramsey RESET test found that we did not misestimate our model by forgetting to use squared terms. Finally, our Jarque-Bera test found normal distributions in our model’s residual terms.

**Fig 6:**
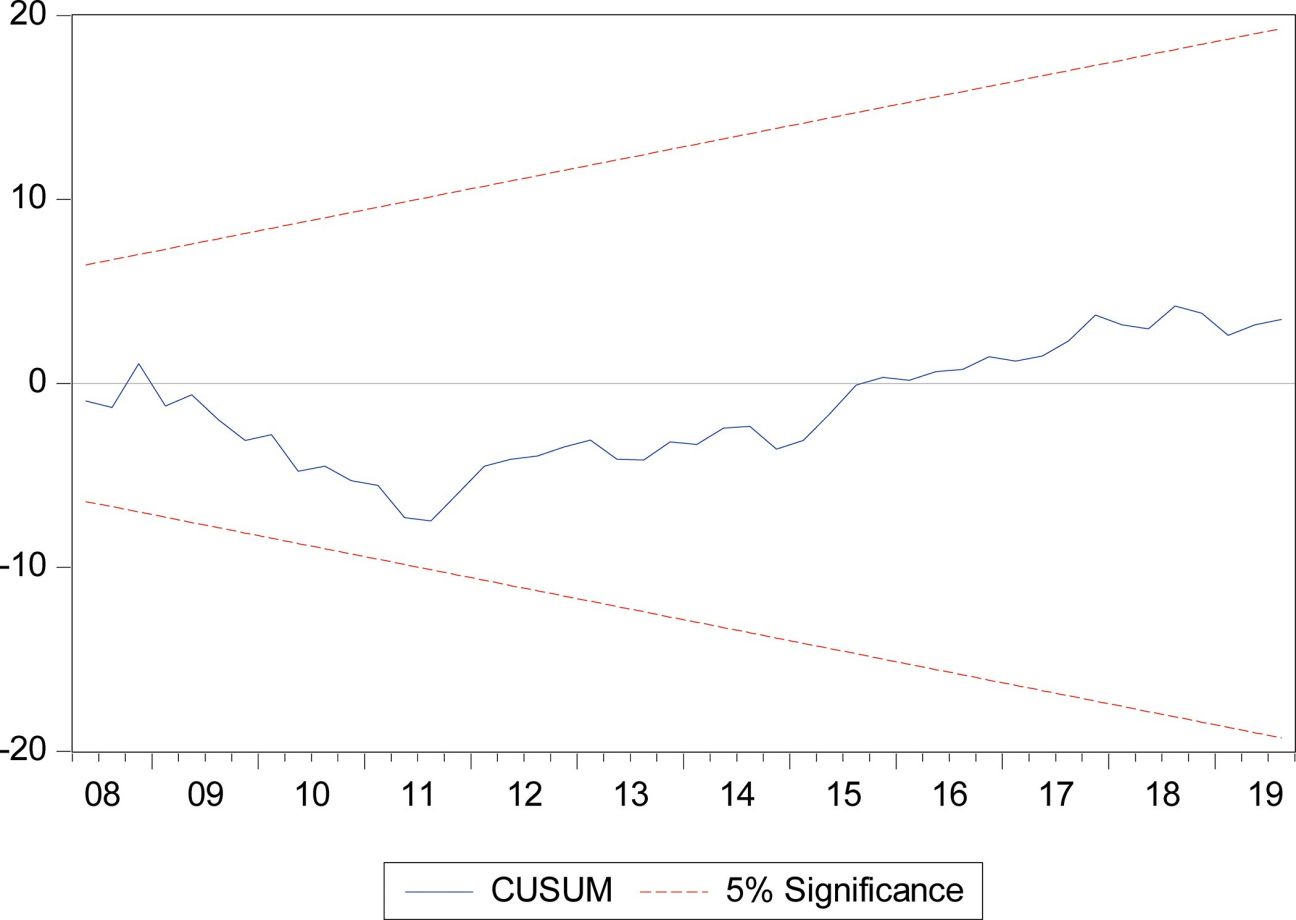
CUSUM test.

**Fig 7:**
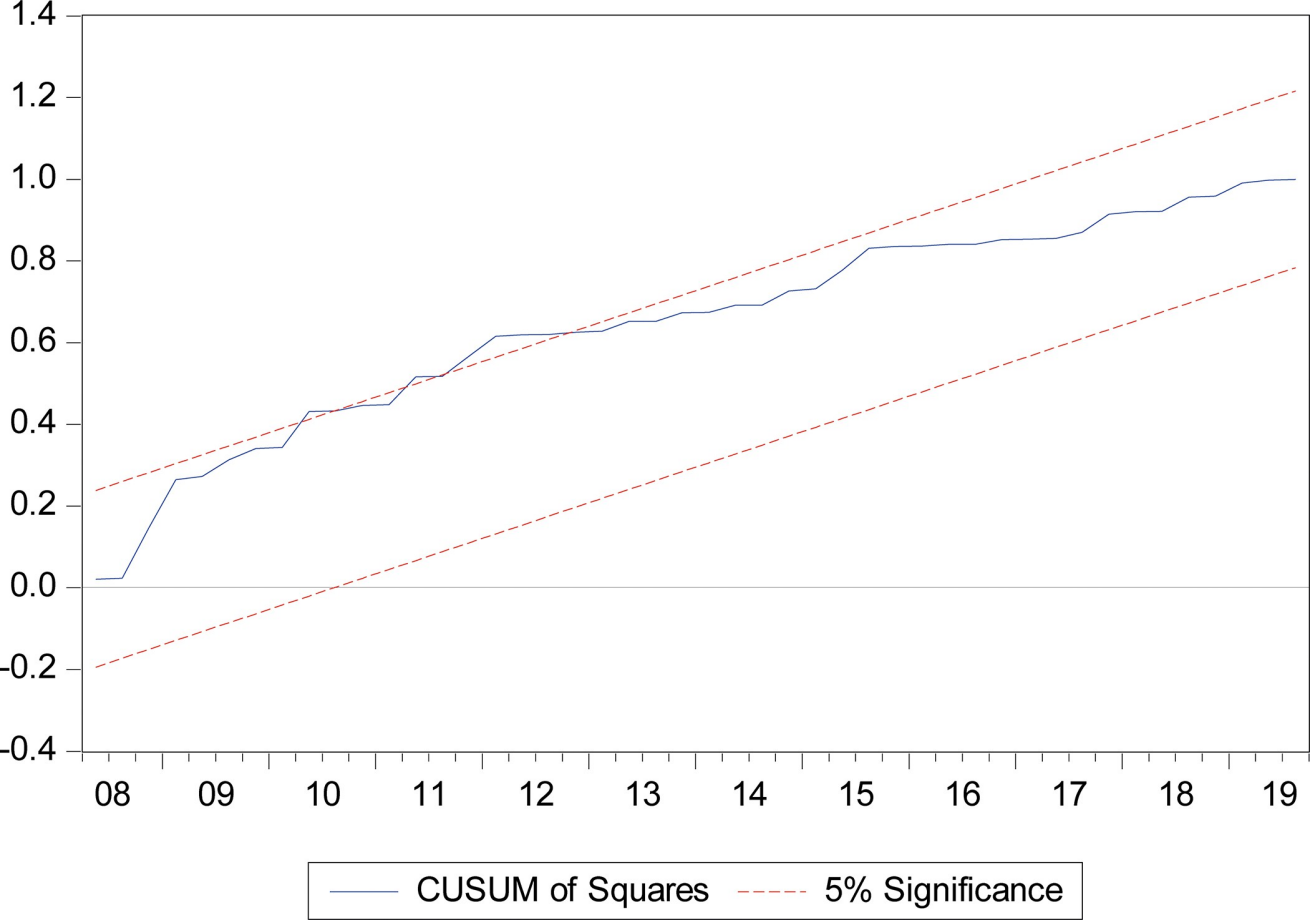
CUSUM of squares test.

Further, the stability of coefficients is tested by CUSUM and CUSUMSQ. The plots of both CUSUM and CUSUMSQ are presented in Figs 6 and 7, respectively. Our results indicate that the estimated CUSUM and CUSUMSQ are generally within the 5 percent significance level, showing that the residual variance is reasonably stable. Finally, we dropped the variable–human capital development *h* in the equation to perform a robustness check. After conducting the robustness check, we found that the coefficients are plausible and robust.

## 6. Conclusions and policy implications

Our work has developed on TALC modeling, mainly how tourism development affects a global city’s economic growth. This study has also examined how tourism development in Hong Kong has affected the city’s economic growth. We investigated the short- and long-run tourism-growth relationship using a dataset from 2003 to 2019 and the ARDL model. We found that Hong Kong likely wrung out most of its tourism industry’s benefits. In the short run, tourism development in Hong Kong spurred the city’s economic growth after a one-quarter lag. We clearly have such lagged effects with introducing tourism-friendly policies, like the visa liberalization policy. Yet, the relatively small impact of tourism on economic growth dissipates away quickly.

Our findings have two significant policy implications. First, Hong Kong’s economic, social, and environmental issues swamp the effects on GDP growth of any pro-tourism policy. Our regression coefficients continued to show that the good old factors of production enshrined in a standard Cobb-Douglas production function affected GDP growth more than tourism policy. The sudden closure of borders between Hong Kong and other countries after 2019 has seriously reduced the number of international tourist arrivals into Hong Kong. Such a sudden stop in tourism has given policymakers ample opportunity to reassess tourism’s role in Hong Kong’s future development. Hong Kong’s future tourism policy should focus on quality above quantity. Mainland visitors will continue representing the lion’s share of Hong Kong’s tourists. Mainland tourism-related firms, government bodies, and industry associations have started focusing tourism activities on higher value-added and productive experiences for all parties.

Second, tourism affects local economies differently over time. The initial positive short-term effects on growth can quickly dissipate away as fundamental factors of production decide GDP growth in the longer term. The 2003 IVS boosted tourism, benefitting Hong Kong’s economic development by fostering employment, increasing tax revenues, and generating positive spillover effects across industries. After almost two decades, though, visa liberalization has yielded diminishing returns. Echoing Qiu, Fan, Lyu, Lin, and Jenkins’s and Tsai’s views on such tourism [9, 35], policymakers may need to reevaluate the equity-efficiency trade-off inherent in tourism development. Using tourism simply to foster economic growth will need to give way to a more sustainable view of tourism.

Our results suggest that tourism development can stimulate economic growth in global cities like Hong Kong. The COVID-19 pandemic may affect how previous tourism policy and planning influenced the global city’s economic growth. However, having econometric estimates of the short-term and long-term relationship between tourism and economic growth can help policymakers develop better tourism strategies. Future research on other global cities like Beijing, Shanghai, Singapore, New York, and London may lead to more specific findings.

## Supporting information

S1 Data (XLSX)
